# Rising trends in the predicted 10-year risk of cardiovascular diseases among Royal Thai Army personnel from 2017 to 2021

**DOI:** 10.1038/s41598-023-28494-3

**Published:** 2023-01-26

**Authors:** Boonsub Sakboonyarat, Jaturon Poovieng, Kanlaya Jongcherdchootrakul, Phutsapong Srisawat, Panadda Hatthachote, Mathirut Mungthin, Ram Rangsin

**Affiliations:** 1 Department of Military and Community Medicine, Phramongkutklao College of Medicine, Bangkok, 10400 Thailand; 2 Department of Medicine, Phramongkutklao College of Medicine, Bangkok, 10400 Thailand; 3 Department of Physiology, Phramongkutklao College of Medicine, Bangkok, 10400 Thailand; 4 Department of Parasitology, Phramongkutklao College of Medicine, Bangkok, 10400 Thailand

**Keywords:** Cardiology, Diseases, Medical research, Risk factors

## Abstract

Deaths from cardiovascular diseases (CVD) are becoming a growing threat to global health, including in Thailand. The aim of the present study was to identify the recent trends in the predicted 10-year risk of CVD among Royal Thai Army (RTA) personnel from 2017 to 2021. The predicted 10-year risk for CVD was calculated through the use of the 2008 updated version of the risk algorithm derived from the Framingham Heart Study data. The current study included 346,355 active-duty RTA personnel aged 30–60 years. The age- and sex-adjusted mean of the predicted 10-year risk for CVD significantly increased from 10.8% (95% CI: 10.8–10.9%) in 2017 to 11.7% (95% CI: 11.6–11.7%) in 2021 (*p* for trend < 0.001). The overall age- and sex-adjusted prevalence of intermediate-to-high predicted 10-year risk for CVD remarkably surged from 24.9% (95% CI: 24.4–25.4%) in 2017 to 29.5% (95% CI: 29.0–30.0%) in 2021 (*p* for trend < 0.001). The modifiable risk factors for CVD, including high systolic blood pressure, high body mass index, and current smoking in this population, should be alleviated to mitigate the risk for CVD in the future.

## Introduction

According to the Global Burden of Disease Study 2017, deaths from cardiovascular diseases (CVD) are becoming more of a threat to global health^1^. Similarly, approximately 75% of all deaths in Thailand resulted from noncommunicable diseases (NCDs), with CVD accounting for one-fourth of that percentage^2^.

Currently, several studies have reported the predicted risk for CVD based on various assessment tools, such as pooled cohort equations and the risk algorithm derived from the Framingham Heart Study (FHS)^3,4^. However, each algorithm had different participant components and endpoints for calculating the predicted risk for CVD^3–5^. For instance, the pooled cohort equation included 24,626 participants (11,381 women) aged 40–79 years and followed up for at least 12 years; the endpoint comprised hard atherosclerotic CVD events (nonfatal myocardial infarction, coronary artery disease death, or fatal or nonfatal stroke)^3,6^. On the contrary, the 2008 updated version of FHS data included 8491 participants (4522 women) aged 30–74 years and followed up for 12 years; the endpoint comprised 10-year CVD events (coronary heart disease, stroke, peripheral artery disease, or heart failure)^4^.

A recent study on U.S. adults demonstrated that the mean predicted risk for CVD based on an algorithm from FHS data was 9.2% from 1999 to 2000 and 8.7% from 2009 to 2010^7^. In Thailand, one study with a small sample size reported that the prevalence of intermediate-to-high predicted 10-year risk for CVD among Thai adults was 51.4% and 28.7% among males and females, respectively^8^. Nevertheless, there was no information on the secular trends in the predicted 10-year risk of CVD in Thailand, among both civilians and military personnel.

Nationwide, nearly 70,000 Royal Thai Army (RTA) personnel aged at least 30 years participate in yearly health examinations provided by the RTA Medical Department (RTAMED). A recent study on RTA personnel demonstrated a considerable increase in the average BMI and other lifestyle factors, including cigarette smoking, from 2017 to 2021 among this population^9–11^. Understanding the trends in the predicted risk for CVD among RTA personnel may help in developing health policies for targeted interventions in order to lessen CVD risk in this population. Therefore, the investigators aimed to determine the recent trends in the predicted 10-year risk for CVD among RTA personnel from 2017 to 2021.

## Methods

### Study design and subjects

**Figure 1:**
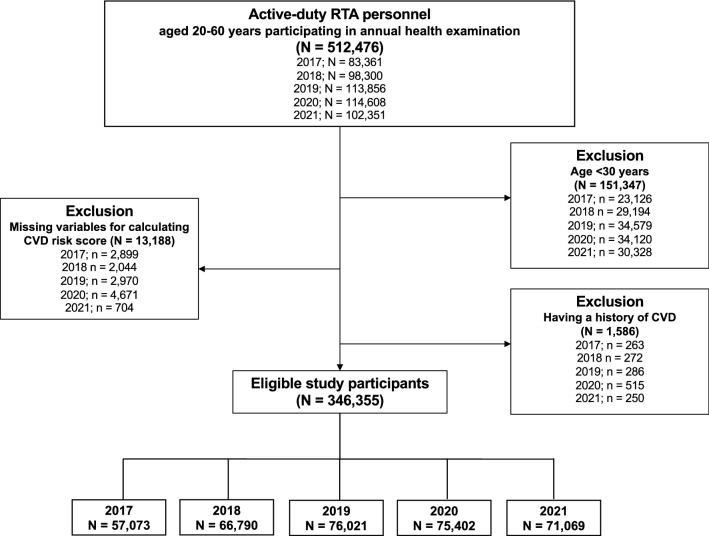
Flowchart of the study.

The RTAMED provides annual health examinations for RTA personnel through 37 RTA hospitals nationwide in Thailand, the Armed Forces Research Institute of Medical Sciences, and the Army Institute of Pathology^9,10^. Such data are reported to the RTAMED in Bangkok, Thailand. We performed a serial cross-sectional study from 2017 to 2021 using the dataset from the annual health examination database of RTA personnel after obtaining permission from the RTAMED. The inclusion criteria for the present study included active-duty RTA personnel aged 30–60 years without a self-reported history of CVD. Since we utilized the collected data, the data for RTA personnel who did not participate in each annual health examination were excluded from the data for that year. A total of 346,355 RTA personnel from 2017 to 2021 were eligible for the current study. The flow of the study is presented in Fig. 1.

### Data collection

A self-report data collection was utilized for obtaining demographic characteristics and comorbidities, including age, sex, health scheme, smoking status, a history of diabetes, and a history of hypertension (HT). Using the data from the responses to the questionnaire, current smoking status was defined as having a history of cigarette smoking within the last 12 months. Self-reported history of CVD was defined as having a history of coronary heart disease, myocardial infarction, heart failure, or stroke. The annual health examination dataset also comprised anthropometric weight, height, and blood pressure (BP) measurements. BMI was calculated as body weight in kilograms divided by height in meters squared: (kg)/(m)^12^. BP was measured using an automatic blood pressure monitor by an operator trained in the standardized technique following the Thai guidelines on treating HT^13^.

### Study outcome

Recently, a related systematic review demonstrated the benefit of global cardiovascular risk assessment in primarily preventing CVD, with most interventions utilizing Framingham or a Framingham-derived risk score^14^. Moreover, SBP (− 4.9 mmHg; 95% CI: − 8.6, − 1.2 mmHg) alleviated, and quitting smoking (risk ratio 1.5; 95% CI: 1.1–2.2) was observed when the CVD risk score information was revealed to patients^14^. Therefore, in the current study, we utilized simple office-based nonlaboratory predicted 10-year risk for CVD, which was calculated using the 2008 updated version of the risk algorithm obtained from the FHS data among individuals without a self-reported history of CVD^4,6^. The CVD algorithm for predicting individual CVD events (coronary heart disease, stroke, peripheral artery disease, or heart failure) included age, sex, BMI, SBP, a history of diabetes, and treatment for HT^4^. The 10-year risk of CVD was classified into three classes as follows: low risk (< 10%), intermediate risk (10–20%), and high risk (> 20%)^4^.

### Statistical analysis

In the present study, the frequency distribution of demographic characteristics was calculated in order to describe the study subjects. Categorical data, including sex, regions, health schemes, smoking status, a history of diabetes, and history of HT, were presented as percentages. Continuous variables, including age, BMI, and SBP, were presented as mean and standard deviation (SD). The age- and sex-adjusted means or proportions of predicted 10-year risk for CVD were calculated using data for each year from 2017 to 2021. Linear regression was employed for age- and sex-adjusted means and logistic regression for proportions in order to test the statistical significance of linear and nonlinear trends from 2017 to 2021. The nonlinear trend was tested first by the addition of a quadratic term to the regression model. If the result was not significant, a linear trend was tested. All analyses were conducted through the use of StataCorp. 2021, *Stata Statistical Software: Release 17*, College Station, TX: StataCorp LLC. All statistical tests were two-sided, and a *p*-value less than 0.05 was considered statistically significant.

### Ethics considerations

The study was reviewed and approved by the Institutional Review Board, Royal Thai Army Medical Department (Approval No. S067h/64_Exp & S056h/65_Exp), in accordance with international guidelines, including the Declaration of Helsinki, the Belmont Report, CIOMS Guidelines, and the International Conference on Harmonization of Technical Requirements for Registration of Pharmaceuticals for Human Use-Good Clinical Practice (ICH-GCP). As a result of utilizing secondary data, a waiver of documentation of informed consent was obtained. The Institutional Review Board of the Royal Thai Army Medical Department approved an informed consent waiver.

## Results

### Demographic characteristics

A total of 346,355 RTA personnel aged 30–60 without a history of CVD were included in the present study. Table 1 presents the demographic characteristics of the participants. The mean age of study participants was in the range of 42.6–44.2 years. Approximately 90% of the participants were males. The percentage of participants residing in central regions ranged from 34.5 to 38.5%. Furthermore, the current smoking rate among RTA personnel has steadily increased from 26.0% in 2017 to 30.5% in 2021. Over 5 years, the average BMI among study participants slightly rose from 25.1 to 25.3 kg/m^2^. Likewise, the average SBP increased from 129.3 mmHg in 2017 to 130.7 mmHg in 2021.

**Table 1:** Characteristics of participants from 2017 to 2021 (N = 346,355). SD; standard deviation.

Year	2017	2018	2019	2020	2021
Characteristics	N = 57,073	N = 66,790	N = 76,021	N = 75,402	N = 71,069
Age, mean (SD), y	44.2 (9.2)	43.6 (9.3)	43.4 (9.3)	43.4 (9.5)	42.6 (9.3)
Sex					
Male, No. (%)	51,949 (91.0)	60,025 (89.9)	68,761 (90.5)	67,000 (88.9)	64,628 (90.9)
Female, No. (%)	5124 (9.0)	6765 (10.1)	7260 (9.5)	8402 (11.1)	6441 (9.1)
Regions					
Bangkok, No. (%)	8780 (15.4)	12,167 (18.2)	13,324 (17.5)	13,788 (18.3)	6813 (9.6)
Central, No. (%)	20,054 (35.1)	23,935 (35.8)	26,253 (34.5)	26,255 (34.8)	27,329 (38.5)
Northeast, No. (%)	10,308 (18.1)	11,774 (17.6)	13,265 (17.4)	15,235 (20.2)	12,760 (18.0)
North, No. (%)	13,302 (23.3)	10,266 (15.4)	16,278 (21.4)	12,607 (16.7)	15,779 (22.2)
South, No. (%)	4629 (8.1)	8648 (12.9)	6901 (9.1)	7517 (10.0)	8388 (11.8)
Health scheme					
Civil Servant Medical Benefit, No. (%)	55,886 (97.9)	65,401 (97.9)	74,581 (98.1)	73,359 (97.3)	69,791 (98.2)
Social Security, No. (%)	775 (1.4)	778 (1.2)	908 (1.2)	1509 (2.0)	1108 (1.6)
Universal Coverage, No. (%)	412 (0.7)	611 (0.9)	532 (0.7)	534 (0.7)	170 (0.2)
Current smoker, No. (%)	14,812 (26.0)	19,302 (28.9)	22,072 (29.0)	23,545 (31.2)	21,685 (30.5)
History of diabetes, No. (%)	8587 (15.0)	9052 (13.6)	10,450 (13.7)	10,559 (14.0)	9914 (13.9)
History of hypertension, No. (%)	8857 (15.5)	9307 (13.9)	10,340 (13.6)	11,571(15.4)	10,842(15.3)
Body mass index, mean (SD), kg/m^2^	25.1 (3.7)	25.1 (3.7)	25.2 (3.8)	25.2 (3.8)	25.3 (3.8)
Systolic blood pressure, mean (SD), mmHg	129.3 (16.5)	129.5 (16.5)	129.6 (16.3)	129.9 (16.2)	130.7 (16.6)

### Trends in the mean predicted 10-year risk for cardiovascular diseases from 2017 to 2021

**Figure 2:**
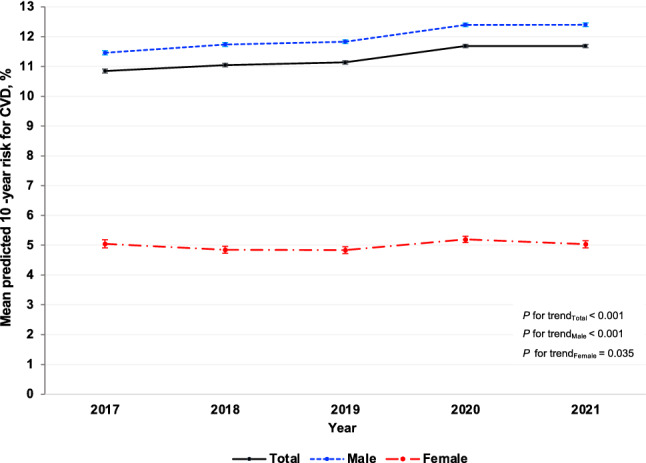
Age-adjusted mean (%) of the predicted 10-year risk for cardiovascular diseases (CVD) among Royal Thai Army personnel stratified by sex from 2017 to 2021.

**Figure 3:**
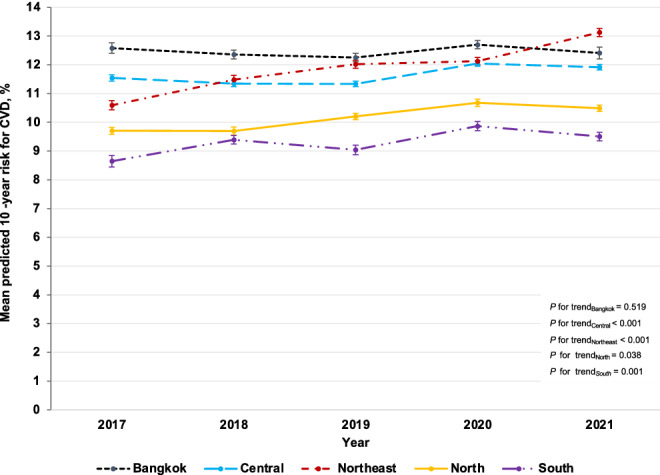
Age- and sex-adjusted mean (%) of the predicted 10-year risk for cardiovascular diseases (CVD) among Royal Thai Army personnel stratified by region from 2017 to 2021.

Table 2 presents the age- and sex-adjusted mean of the predicted 10-year risk for CVD among RTA personnel from 2017 to 2021. The age- and sex-adjusted mean of the predicted 10-year risk for CVD significantly increased from 10.8% (95% CI: 10.8–10.9%) in 2017 to 11.7% (95% CI: 11.6–11.7%) in 2021 (*p* for trend < 0.001). The age-adjusted mean of the predicted 10-year risk for CVD among males rose from 11.5% (95% CI: 11.4–11.5%) in 2017 to 12.4 (95% CI: 12.3–12.5%) in 2021 (*p* for trend < 0.001). Meanwhile, the age-adjusted mean of the predicted 10-year risk for CVD among females was consistent over 5 years, accounting for 5.0% (95% CI: 4.9–5.2%) in 2017 and 2021 (Fig. 2). As regards age groups, the sex-adjusted mean of the predicted 10-year risk for CVD among RTA personnel significantly increased over 5 years in all age categories (Table 2). Particularly, it continuously increased among those aged $\ge$ 55 years from 22.5% (95% CI: 22.3–22.8%) in 2017 to 24.7% (95% CI: 24.4–24.9%) in 2021 (*p* for trend < 0.001). Figure 3 illustrates the age- and sex-adjusted mean of the predicted 10-year risk for CVD among RTA personnel from 2017 to 2021, stratified by regions. The age- and sex-adjusted mean of the predicted 10-year risk for CVD among study participants residing in the northeast significantly surged from 10.6% (95% CI: 10.4–10.8%) in 2017 to 13.1% (95% CI: 13.0–13.3%) in 2021 (*p* for trend < 0.001). Meanwhile, the age- and sex-adjusted mean of the predicted 10-year risk for CVD among RTA personnel residing in central, north, and south slightly increased over 5 years (*p* for trend < 0.05). However, the age- and sex-adjusted mean predicted 10-year risk for CVD among participants in Bangkok was consistent over 5 years (*p* for trend = 0.519) (Table 2).

**Table 2:** Means (%) of predicted 10-year risk for CVD among RTA personnel from 2017 to 2021.

Year	2017			2018			2019			2020			2021			*p* for trend
	N	Mean	95% CI	N	Mean	95% CI	N	Mean	95% CI	N	Mean	95% CI	N	Mean	95% CI	
Total^a^	57,073	10.8	10.8–10.9	66,790	11.0	11.0–11.1	76,021	11.1	11.1–11.2	75,402	11.7	11.6–11.7	71,069	11.7	11.6–11.7	< 0.001
Sex^b^																
Male	51,949	11.5	11.4–11.5	60,025	11.7	11.7–11.8	68,761	11.8	11.8–11.9	67,000	12.4	12.3–12.5	64,628	12.4	12.3–12.5	< 0.001
Female	5124	5.0	4.9–5.2	6765	4.8	4.7–5.0	7260	4.8	4.7–5.0	8402	5.2	5.1–5.3	6441	5.0	4.9–5.2	0.035
Age (years)^c^																
30–34	12,656	3.1	3.1–3.1	16,099	3.3	3.2–3.3	18,821	3.3	3.3–3.3	18,783	3.4	3.4–3.4	18,918	3.5	3.5–3.5	< 0.001
35–39	8100	5.3	5.2–5.3	10,107	5.3	5.2–5.3	12,015	5.3	5.2–5.3	12,839	5.3	5.2–5.3	13,720	5.5	5.4–5.5	< 0.001^d^
40–44	8152	8.3	8.2–8.4	9968	8.5	8.4–8.7	11,167	8.7	8.6–8.8	10,191	8.6	8.5–8.7	9415	9.0	8.9–9.1	< 0.001
45–49	6956	12.2	12.0–12.4	7902	12.5	12.3–12.6	8872	12.5	12.3–12.7	9283	12.8	12.6–13.0	8955	12.9	12.7–13.1	< 0.001
50–54	10,914	17.3	17.2–17.5	11,113	17.4	17.2–17.5	11,707	17.5	17.3–17.7	10,323	18.5	18.3–18.7	8442	18.4	18.1–18.6	< 0.001
55–60	10,295	22.5	22.3–22.8	11,601	23.1	22.9–23.4	13,439	23.1	22.9–23.3	13,983	24.8	24.6–25.1	11,619	24.7	24.4–24.9	< 0.001
Regions^a^																
Bangkok	8780	12.6	12.4–12.8	12,167	12.4	12.2–12.5	13,324	12.3	12.1–12.4	13,788	12.7	12.6–12.8	6813	12.4	12.2–12.6	0.519
Central	20,054	11.5	11.4–11.7	23,935	11.3	11.2–11.4	26,253	11.3	11.2–11.4	26,255	12.0	11.9–12.1	27,329	11.9	11.8–12.0	< 0.001^d^
Northeast	10,308	10.6	10.4–10.8	11,774	11.5	11.3–11.6	13,265	12.0	11.9–12.2	15,235	12.1	12.0–12.3	12,760	13.1	13.0–13.3	< 0.001
North	13,302	9.7	9.6–9.8	10,266	9.7	9.6–9.8	16,278	10.2	10.1–10.3	12,607	10.7	10.6–10.8	15,779	10.5	10.4–10.6	0.038^d^
South	4629	8.6	8.4–8.8	8648	9.4	9.2–9.5	6901	9.0	8.9–9.2	7517	9.9	9.7–10.0	8388	9.5	9.4–9.7	0.001^d^

^a^Age- and sex adjusted mean using regression analyses.

^b^Age-adjusted mean using regression analyses.

^c^Sex-adjusted mean using regression analyses.

^d^Nonlinear trend.

*CVD* cardiovascular diseases, *RTA* Royal Thai Army, *95% CI* 95% confidence interval.

### Trends in the prevalence of intermediate-to-high predicted 10-year risk for cardiovascular diseases from 2017 to 2021

**Figure 4:**
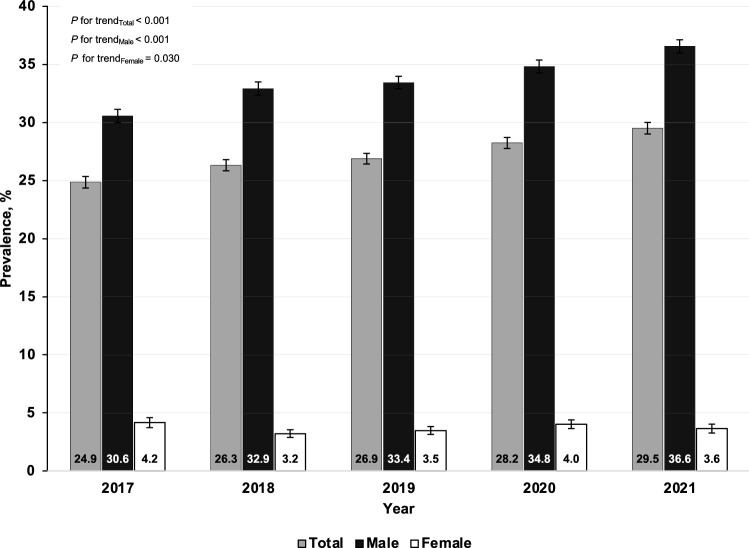
Age-adjusted prevalence (%) of the intermediate-to-high predicted 10-year risk for cardiovascular diseases (CVD) among Royal Thai Army personnel stratified by sex from 2017 to 2021.

**Figure 5:**
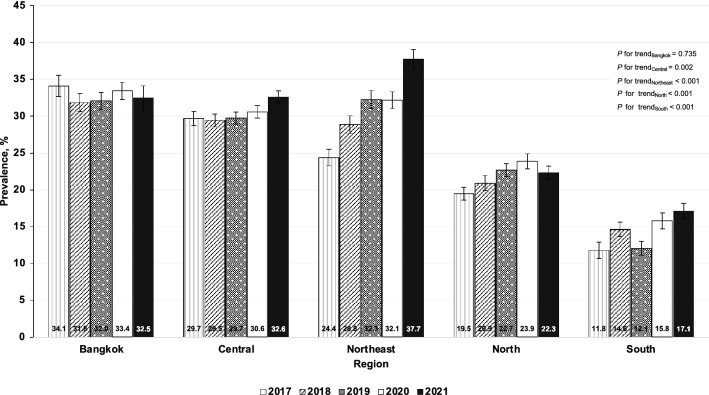
Age- and sex-adjusted prevalence (%) of the intermediate-to-high predicted 10-year risk for cardiovascular diseases (CVD) among Royal Thai Army personnel stratified by region from 2017 to 2021.

Table 3 shows the age- and sex-adjusted prevalence of intermediate-to-high predicted 10-year risk for CVD among RTA personnel from 2017 to 2021. The overall age- and sex-adjusted prevalence of intermediate-to-high predicted 10-year risk for CVD considerably increased from 24.9% (95% CI: 24.4–25.4%) in 2017 to 29.5% (95% CI: 29.0–30.0%) in 2021 (*p* for trend < 0.001). The age-adjusted prevalence of intermediate-to-high predicted 10-year risk for CVD continuously increased among males from 30.6% (95% CI: 30.0–31.1%) in 2017 to 36.6% (95% CI: 36.0–37.1%) in 2021 (*p* for trend < 0.001). On the other hand, the age-adjusted prevalence of intermediate-to-high predicted 10-year risk for CVD among females was in the range of 3.2–4.2% over 5 years (*p* for trend 0.030) (Fig. 4). As regards age groups, the sex-adjusted prevalence of intermediate-to-high predicted 10-year risk for CVD among RTA personnel remarkably rose over 5 years in all age categories (*p* for trend < 0.05) (Table 3). Figure 5 illustrates the age- and sex-adjusted prevalence of intermediate-to-high predicted 10-year risk for CVD among RTA personnel from 2017 to 2021, stratified by regions. The age- and sex-adjusted prevalence of intermediate-to-high predicted 10-year risk among RTA personnel residing in the northeast significantly increased from 24.4% (95% CI: 23.2–25.5%) in 2017 to 37.7% (95% CI: 36.4–39.0%) in 2021, which was the highest increase in comparison with those in other regions (*p* for trend < 0.001) (Table 3). In Bangkok, the age- and sex-adjusted prevalence of intermediate-to-high predicted 10-year risk for CVD was consistently high over 5 years (*p* for trend = 0.735), being 34.1 in 2017 and 32.5 in 2021. Rising trends in the age- and sex-adjusted prevalence of intermediate-to-high predicted 10-year risk among RTA personnel residing in other regions, except Bangkok, over 5 years were also observed (*p* for trend < 0.05) (Table 3).

**Table 3:** Prevalence of intermediate-to-high of predicted 10-year risk for CVD among RTA personnel from 2017 to 2021.

Year	2017			2018			2019			2020			2021			*p* for trend
	N	%	95% CI	N	%	95% CI	N	%	95% CI	N	%	95% CI	N	%	95% CI	
Total^a^	57,073	24.9	24.4–25.4	66,790	26.3	25.8–26.8	76,021	26.9	26.4–27.3	75,402	28.2	27.8–28.7	71,069	29.5	29.0–30.0	< 0.001
Sex^b^																
Male	51,949	30.6	30.0–31.1	60,025	32.9	32.4–33.5	68,761	33.4	32.9–34.0	67,000	34.8	34.3–35.4	64,628	36.6	36.0–37.1	< 0.001
Female	5124	4.2	3.7–4.6	6765	3.2	2.9–3.6	7260	3.5	3.1–3.9	8402	4.0	3.7–4.4	6441	3.6	3.3–4.1	0.030^d^
Age (years)^c^																
30–34	12,656	0.4	0.3–0.5	16,099	0.6	0.5–0.7	18,821	0.7	0.6–0.8	18,783	0.7	0.6–0.9	18,918	1.1	0.9–1.3	< 0.001
35–39	8100	6.6	6.1–7.2	10,107	6.1	5.6–6.6	12,015	6.3	5.8–6.8	12,839	6.0	5.5–6.4	13,720	7.1	6.6–7.6	< 0.001
40–44	8152	23.4	22.5–24.4	9968	26.0	25.1–26.9	11,167	26.2	25.3–27.1	10,191	26.4	25.5–27.3	9415	28.0	27.1–29.0	< 0.001
45–49	6956	45.0	43.8–46.2	7902	47.6	46.4–48.8	8872	47.3	46.2–48.5	9283	49.2	48.1–50.3	8955	49.7	48.6–50.8	< 0.001
50–54	10,914	76.4	75.5–77.2	11,113	76.9	76.1–77.8	11,707	76.4	75.5–77.2	10,323	78.7	77.8–79.5	8442	79.3	78.4–80.3	0.040^d^
55–60	10,295	94.3	93.9–94.8	11,601	94.3	93.9–94.7	13,439	94.9	94.5–95.2	13,983	95.5	95.1–95.8	11,619	95.4	95.0–95.8	< 0.001
Regions^a^																
Bangkok	8780	34.1	32.6–35.6	12,167	31.8	30.6–33.0	13,324	32.0	30.9–33.2	13,788	33.4	32.3–34.6	6813	32.5	30.9–34.2	0.735
Central	20,054	29.7	28.7–30.6	23,935	29.5	28.6–30.3	26,253	29.7	28.9–30.6	26,255	30.6	29.7–31.4	27,329	32.6	31.8–33.4	0.002^d^
Northeast	10,308	24.4	23.2–25.5	11,774	28.8	27.7–30.1	13,265	32.3	31.1–33.5	15,235	32.1	31.0–33.3	12,760	37.7	36.4–39.0	< 0.001
North	13,302	19.5	18.6–20.4	10,266	20.9	19.9–22.0	16,278	22.7	21.8–23.6	12,607	23.9	22.9–24.9	15,779	22.3	21.4–23.2	< 0.001
South	4629	11.8	10.7–13.0	8648	14.6	13.7–15.7	6901	12.1	11.1–13.1	7517	15.8	14.7–16.9	8388	17.1	16.0–18.2	< 0.001

^a^Age- and sex adjusted prevalence using regression analyses.

^b^Age-adjusted prevalence using regression analyses.

^c^Sex-adjusted prevalence using regression analyses.

^d^Nonlinear trend.

*CVD* cardiovascular diseases, *RTA* Royal Thai Army, *95% CI* 95% confidence interval.

### Trends in the prevalence of high predicted 10-year risk for cardiovascular diseases from 2017 to 2021

Table 4 shows the age- and sex-adjusted prevalence of the high predicted 10-year risk for CVD among RTA personnel from 2017 to 2021. The overall age- and sex-adjusted prevalence of high predicted 10-year risk for CVD remarkably rose from 4.9% (95% CI: 4.8–5.1%) in 2017 to 5.8% (95% CI: 5.6–5.9%) in 2021 (*p* for trend < 0.001). The age-adjusted prevalence of high predicted 10-year risk for CVD among males increased from 5.9% (95% CI: 5.7–6.1%) in 2017 to 6.1% (95% CI: 6.0–6.3%) in 2018, reached 7.2% (95% CI: 7.0–7.4%) in 2020, and then slightly dropped to 7.0% (95% CI: 6.8–7.2%) in 2021 (*p* for trend < 0.001). Meanwhile, the age-adjusted prevalence of the high predicted 10-year risk for CVD among females was relatively low, ranging from 0.6 to 0.8% over 5 years (*p* for a trend of 0.578). Over a half-decade, rising trends in the age- and sex-adjusted prevalence of high predicted 10-year risk for CVD among RTA personnel aged at least 35 years were observed. In particular, it significantly surged among RTA personnel aged 55 years or more from 45.3% (95% CI: 44.3–46.3%) in 2017 to 53.4% (95% CI: 52.6–54.3%) in 2020. Then, it slightly dropped to 52.1% (95% CI: 51.1–53.0%) in 2021 (*p* for trend < 0.001). Regarding regions, the age- and sex-adjusted prevalence of high predicted 10-year risk for CVD among study participants in Bangkok was steadily high, ranging from 7.2 to 8.1% over 5 years. On the other hand, the age- and sex-adjusted prevalence of high predicted 10-year risk for CVD among those residing in other regions significantly increased (*p* for trend < 0.05); especially in the northeast, it rose from 5.0% (95% CI: 4.7–5.4%) in 2017 to 8.4% (95% CI: 8.0–9.0%) in 2021 (*p *for trend < 0.001).

**Table 4:** Prevalence of high predicted 10-year risk for CVD among RTA personnel, from 2017 to 2021.

	2017			2018			2019			2020			2021			*p* for trend
	N	%	95% CI	N	%	95% CI	N	%	95% CI	N	%	95% CI	N	%	95% CI	
Total^a^	57,073	4.9	4.8–5.1	66,790	5.1	4.9–5.2	76,021	5.1	5.0–5.3	75,402	6.0	5.8–6.1	71,069	5.8	5.6–5.9	< 0.001
Sex^b^																
Male	51,949	5.9	5.7–6.1	60,025	6.1	6.0–6.3	68,761	6.2	6.0–6.4	67,000	7.2	7.0–7.4	64,628	7.0	6.8–7.2	< 0.001
Female	5124	0.7	0.6–0.9	6765	0.6	0.5–0.7	7260	0.6	0.5–0.7	8402	0.8	0.6–0.9	6441	0.6	0.5–0.8	0.578^**d**^
Age (years)^c^																
30–34	12,656	N/A	N/A	16,099	N/A	N/A	18,821	N/A	N/A	18,783	N/A	N/A	18,918	N/A	N/A	N/A
35–39	8100	0.7	0.5–0.9	10,107	0.5	0.4–0.7	12,015	0.6	0.4–0.8	12,839	0.4	0.3–0.6	13,720	0.6	0.5–0.8	0.029^**d**^
40–44	8152	3.6	3.2–4.1	9968	3.6	3.2–4.0	11,167	4.1	3.7–4.5	10,191	3.8	3.4–4.2	9415	4.9	4.4–5.4	< 0.001
45–49	6956	12.1	11.3–12.9	7902	12.3	11.6–13.1	8872	12.6	11.9–13.3	9283	13.4	12.7–14.1	8955	13.7	13.0–14.5	< 0.001
50–54	10,914	28.8	27.9–29.6	11,113	28.9	28.0–29.7	11,707	29.6	28.7–30.4	10,323	34.2	33.3–35.2	8442	32.3	31.3–33.3	< 0.001
55–60	10,295	45.3	44.3–46.3	11,601	48.0	47.0–48.9	13,439	47.3	46.4–48.2	13,983	53.4	52.6–54.3	11,619	52.1	51.1–53.0	< 0.001
Regions^a^																
Bangkok	8780	8.0	7.4–8.5	12,167	7.2	6.8–7.7	13,324	7.4	7.0–7.9	13,788	8.1	7.6–8.6	6813	7.8	7.2–8.4	0.246
Central	20,054	6.1	5.8–6.4	23,935	5.6	5.4–5.9	26,253	5.4	5.2–5.7	26,255	7.0	6.7–7.3	27,329	6.2	6.0–6.5	0.005^**d**^
Northeast	10,308	5.0	4.7–5.4	11,774	6.1	5.8–6.5	13,265	6.7	6.3–7.2	15,235	6.6	6.2–7.0	12,760	8.4	8.0–9.0	< 0.001
North	13,302	3.4	3.2–3.7	10,266	3.3	3.0–3.6	16,278	3.9	3.6–4.1	12,607	4.6	4.3–4.9	15,779	4.1	3.8–4.4	< 0.001
South	4629	1.6	1.4–1.9	8648	2.2	2.0–2.5	6901	1.9	1.7–2.1	7517	2.3	2.1–2.6	8388	2.1	1.8–2.3	0.005^**d**^

^a^Age- and sex adjusted prevalence using regression analyses.

^b^Age-adjusted prevalence using regression analyses.

^c^Sex-adjusted prevalence using regression analyses.

^d^Nonlinear trend.

*CVD* cardiovascular diseases, *RTA* Royal Thai Army, *95% CI* 95% confidence interval.

## Discussion

In the present study, we successfully enrolled 346,355 RTA personnel aged 30–60 years with no history of CVD from 2017 to 2021 nationwide. Rising trends were observed in age- and sex-adjusted predicted 10-year risk of CVD for 5 years. The difference in the age-adjusted predicted 10-year risk of CVD persisted between males and females over a half-decade. Furthermore, the age- and sex-adjusted predicted 10-year risk of CVD among RTA personnel in Bangkok was persistently high, whereas, in other regions, it significantly rose over 5 years, particularly in the northeast.

In Thailand, one related study with a small sample size investigated the predicted 10-year risk of CVD among the general Thai population based on the FHS risk score^8^. A few studies in northeast^15^ and southern^16^ Thailand also examined the predicted 10-year risk of CVD based on the Thai CV risk score. Nevertheless, no information is available on the secular trends in the predicted 10-year risk of CVD in Thailand in both civilian and military personnel. To the best of our knowledge, the current study represented the most extensive epidemiologic study of the trends in the predicted 10-year risk of CVD through the use of the risk algorithm derived from the FHS data among RTA personnel in Thailand.

A study by Putadechakum et al.^8^ demonstrated that the prevalence of intermediate-to-high predicted 10-year risk for CVD among Thai adults was 51.4% and 28.7% among males and females, respectively. We found that, in comparison with this related study in Thailand, the prevalence was relatively low among male and female participants in the present study^8^. The mean predicted 10-year risk of CVD in the present study was comparable to a related study on U.S. adults^7^. Meanwhile, the prevalence of intermediate-to-high predicted 10-year risk of CVD in the present study was lower than that among adults in China^17^.

However, our results indicated that the predicted 10-year risk of CVD in RTA personnel was higher than that among military personnel in other countries^18,19^. For instance, a related study on Iranian military personnel reported that the prevalence of intermediate-to-high predicted 10-year risk of CVD was 2.0% and 1.5% for intermediate and high levels, respectively^19^. On the other hand, the mean predicted 10-year risk of CVD among the militaries in the Kingdom of Saudi Arabia was 4.5 ± 4.2%^18^. This finding may be attributed to the difference in established cardiovascular risk factors, both lifestyle and metabolic risk; for instance, the prevalence of current smokers among RTA personnel was approximately 30%, whereas it was 4.2% among Iranian military personnel^19^. Moreover, the average SBP and prevalence of diabetes among RTA personnel were also higher than those among military personnel in Iran^19^ and Saudi Arabia^18^.

A related study on U.S. adults revealed that the mean predicted 10-year risk of CVD steadily ranged from 8.6 to 9.4% from 1999 to 2010^7^. Conversely, our finding reported that, from 2017 to 2021, the age- and sex-adjusted mean of the predicted 10-year risk of CVD among RTA personnel considerably increased. Furthermore, the rising trends in the prevalence of both intermediate-to-high and high predicted 10-year risk for CVD from 2017 to 2021 were also observed. This finding may result from an increase in lifestyle factors and metabolic risk factors among the study participants over 5 years, such as current smoking, SBP, and BMI, which were utilized for calculating the predicted 10-year risk for CVD. In particular, higher SBP would influence the predicted 10-year risk for CVD based on the algorithm from FHS data^4^. In addition, a recent study also indicated that the obesity prevalence among RTA personnel significantly rose from 2017 to 2021^9^. At the same time, our study reported that the rate of current smokers rose by 5% over 5 years.

In accordance with existing literature^7,18–20^, the higher-age population tended to have a greater risk for CVD. Similarly, our results demonstrated that the individuals of higher age had a higher sex-adjusted 10-year risk for CVD, especially among RTA personnel aged $\ge$ 55. Furthermore, a substantially increasing trend in the sex-adjusted prevalence of the high predicted 10-year risk for CVD was also observed among this age group. Inversely, the related study on U.S. adults indicated a significant reduction in the predicted 10-year risk for CVD among individuals aged $\ge$ 50 from 1999 to 2010.

Our findings revealed that the age-adjusted mean predicted 10-year risk for CVD among male participants was more likely to be higher than that among females over 5 years, which was consistent with the related study on U.S. adults based on the algorithm from FHS data^7^ and the pooled cohort equations ^20^. Moreover, we observed a considerable increase in the age-adjusted prevalence of the high predicted 10-year risk for CVD among male RTA personnel from 2017 to 2021. This finding may be explained by the established lifestyle and metabolic risk factors for CVD among males, which is greater than that among females in Thailand^9,21–23^.

Additionally, we found that the predicted 10-year risk for CVD among RTA personnel residing in Bangkok was still consistently high over 5 years. Concurrently, we observed the significantly rising trends in the predicted 10-year risk for CVD of those in other regions, especially in the northeast. A recent related study may explain these findings among this population, which observed the highest prevalence of obesity among RTA personnel residing in Bangkok and significantly increasing obesity prevalence among RTA personnel residing in other regions, especially in the northeast (41.5% in 2017 to 45.8% in 2021)^9^.

SBP is one of the significant predictors in the FHS risk equation, and the recent increase in this risk factor may contribute to the increased CVD risk among RTA personnel. Moreover, our study demonstrated that the modifiable risk factors for CVD, including smoking and average BMI, among study participants tended to increase over 5 years, probably resulting in an increase in the predicted 10-year risk for CVD among this population. Therefore, our results suggested that the predicted 10-year risk for CVD should be recognized, especially in higher-aged individuals and male RTA personnel. Furthermore, the modifiable lifestyle and metabolic risk factors should be mitigated in order to lower the risk for CVD. For instance, during the annual health examination session, RTA personnel should be advised to reduce or stop smoking, and tobacco cessation support should be provided for RTA personnel^24^. As regards the high BMI, weight management through lifestyle change, including regular exercise and a healthy diet, should be promoted among RTA personnel^25–27^.

In terms of high SBP, lifestyle modifications, including a low salt diet, weight loss, increased physical activity, and alcohol restriction^28–30^, should be encouraged among this population.

The robust evidence indicated that HT treatment strategies based on CVD risk assessment were more effective than one based on BP levels alone. In addition, the related study also demonstrated that the numbers needed to treat for 5 years to avoid one CVD event were lower with the HT treatment strategies based on CVD risk assessment (− 28.5%; 95% CI: − 31.1 to − 25.6%) than with SBP treatment strategies^31^. Therefore, for the RTA personnel with HT, the appropriate pharmacologic therapy and regular physician appointments based on CVD risk assessment should also be performed^30,32,33^.

The present study has several limitations as follows. This study included a series of cross-sectional surveys; longitudinal changes in the risk for CVD at an individual level could not be evaluated. Consequently, the current study calculated the predicted 10-year risk of CVD based on FHS; however, it was based on the FHS data conducted on the U.S. population. Therefore, the validity of CVD risk prediction among Thai people may be relatively inaccurate. Nevertheless, a retrospective cohort study validated the FHS–CVD risk score in a multiethnic Asian population, indicating that the CVD risk prediction score provides fairly accurate predictions for males and somewhat overestimates for females^34^. The current study had considerable strengths, including representing the largest sample of RTA personnel for predicting the 10-year risk for CVD. Thus, our results provided valuable insights into the rising trends in the predicted 10-year risk for CVD of this population.

## Conclusion

This serial cross-sectional study determined the rising trends in the predicted 10-year risk for CVD among RTA personnel from 2017 to 2021. Male RTA personnel are more likely to have a higher predicted 10-year risk for CVD than females over 5 years. The modifiable risk factors for CVD, including high SBP, high BMI, and current smoking among this population, should be alleviated in order to mitigate the risk for CVD in the future.

## Data Availability

The data that support the findings of this study are available from the Royal Thai Army Medical Department, Bangkok, Thailand but restrictions apply to the availability of these data, which were used under license for the current study, and so are not publicly available. Data are however available from the authors upon reasonable request and with permission of the Royal Thai Army Medical Department, Bangkok, Thailand (contact Boonsub Sakboonyarat via boonsub1991@pcm.ac.th).

## Abbreviations

NCDs: Noncommunicable diseases
CVD: Cardiovascular diseases
FHS: Framingham heart study
BMI: Body mass index
SBP: Systolic blood pressure
HT: Hypertension
RTA: The Royal Thai Army
RTAMED: The Royal Thai Army Medical Department
NHES: National health examination survey
CI: Confidence interval
SD: Standard deviation

## References

[1] Global, regional, and national age-sex-specific mortality for 282 causes of death in 195 countries and territories, 1980–2017: A systematic analysis for the Global Burden of Disease Study 2017. *Lancet* **392**, 1736–1788 (2018). 10.1016/S0140-6736(18)32203-7 PMC6227606 30496103

[2] Organization, W. H. Noncommunicable diseases country profiles 2018. (2018).

[3] Zhang Y (2022). Cardiovascular risk assessment tools in Asia. J. Clin. Hypertens.

[4] D’Agostino RB (2008). General cardiovascular risk profile for use in primary care: The Framingham heart study. Circulation.

[5] Duangrithi D, Wattanasermkit R, Rungwijee S, Khunsom N (2020). Thai CV risk score and primary prevention in impaired fasting plasma glucose or diabetes mellitus versus normoglycemia in patients with metabolic syndrome. Int. J. Prev. Med..

[6] Tsao CW, Vasan RS (2015). Cohort profile: The Framingham Heart Study (FHS): Overview of milestones in cardiovascular epidemiology. Int. J. Epidemiol..

[7] Ford ES (2013). Trends in predicted 10-year risk of coronary heart disease and cardiovascular disease among U.S. adults from 1999 to 2010. J. Am. Coll. Cardiol..

[8] Putadechakum, S., Leelahakul, V., Klangjareonchai, T. & Roongpisuthipong, C. Ten-year cardiovascular risk prediction among 194 thai middle-aged adultS. in *Annals of Nutrition and Metabolism* vol. 63 1217 (Karger Allschwilerstrasse 10, CH-4009 Basel, Switzerland, 2013).

[9] Sakboonyarat B (2022). Rising trends in obesity prevalence among Royal Thai Army personnel from 2017 to 2021. Sci. Rep..

[10] Sakboonyarat B (2022). Prevalence of hypertriglyceridemia among Royal Thai Army personnel and its related cardiometabolic risk factors, from 2017 to 2021. BMC Public Health.

[11] Sakboonyarat B, Rangsin R, Mittleman MA (2022). Incidence and risk factors of metabolic syndrome among Royal Thai Army personnel. Sci Rep.

[12] Inoue S (2000). The Asia-Pacific Perspective: Redefining Obesity and Its Treatment.

[13] Kunanon S (2021). 2019 Thai Guidelines on the Treatment of Hypertension: Executive Summary. J. Med. Assoc. Thai..

[14] Collins DRJ (2017). Global cardiovascular risk assessment in the primary prevention of cardiovascular disease in adults: Systematic review of systematic reviews. BMJ Open.

[15] Kingkaew N, Antadech T (2019). Cardiovascular risk factors and 10-year CV risk scores in adults aged 30–70 years old in Amnat Charoen Province, Thailand. Asia-Pac. J. Sci. Technol..

[16] Aramcharoen, S., Satian, P., Chotikarn, P. & Triukose, S. An external validation of Thais’ cardiovascular 10-year risk assessment in the southern Thailand. arXiv:1811.03860 (2018).

[17] Wang YX (2021). Predicted 10-year cardiovascular disease risk and its association with sleep duration among adults in Beijing–Tianjin–Hebei Region, China. Biomed. Environ. Sci.

[18] Al-Dahi S (2013). Assessment of framingham cardiovascular disease risk among militaries in the Kingdom of Saudi Arabia. Mil Med..

[19] Parastouei K, Sepandi M, Eskandari E (2021). Predicting the 10-year risk of cardiovascular diseases and its relation to healthy diet indicator in Iranian military personnel. BMC Cardiovasc. Disord..

[20] He J (2021). Trends in cardiovascular risk factors in US adults by race and ethnicity and socioeconomic status, 1999–2018. JAMA J. Am. Med. Assoc..

[21] Sakboonyarat B, Rangsin R (2018). Prevalence and associated factors of ischemic heart disease (IHD) among patients with diabetes mellitus: A nation-wide, cross-sectional survey. BMC Cardiovasc. Disord..

[22] Soitong P (2021). Association of neck circumference and hypertension among adults in a rural community Thailand: A cross-sectional study. PLoS ONE.

[23] Sakboonyarat B, Rangsin R, Kantiwong A, Mungthin M (2019). Prevalence and associated factors of uncontrolled hypertension among hypertensive patients: A nation-wide survey in Thailand. BMC Res. Notes.

[24] Peer N, Kengne AP (2018). Tobacco cessation in low- and middle-income countries: Some challenges and opportunities. Addiction.

[25] Institute of Medicine. Weight Management: State of the Science and Opportunities for Military Programs. in *Weight Management: State of the Science and Opportunities for Military Programs* (2004).

[26] Ko I-G, Choi P-B (2013). Regular exercise modulates obesity factors and body composition in sturdy men. J. Exerc. Rehabil..

[27] Pan XR (1997). Effects of diet and exercise in preventing NIDDM in people with impaired glucose tolerance: The Da Qing IGT and diabetes study. Diabetes Care.

[28] Todorova B (2004). The G-250A promoter polymorphism of the hepatic lipase gene predicts the conversion from impaired glucose tolerance to type 2 diabetes mellitus: The finnish diabetes prevention study. J. Clin. Endocrinol. Metabol..

[29] Sacks FM (2001). DASH-sodium collaborative research group. Effects on blood pressure of reduced dietary sodium and the Dietary Approaches to Stop Hypertension (DASH) diet. N. Engl. J. Med..

[30] Lastra G, Syed S, Kurukulasuriya LR, Manrique C, Sowers JR (2014). Type 2 diabetes mellitus and hypertension: An update. Endocrinol. Metabol. Clin. N. Am..

[31] Karmali KN (2018). Blood pressure-lowering treatment strategies based on cardiovascular risk versus blood pressure: A meta-analysis of individual participant data. PLoS Med..

[32] Ruggenenti P, Perna A, Ganeva M, Ene-Iordache B, Remuzzi G (2006). Impact of blood pressure control and angiotensin-converting enzyme inhibitor therapy on new-onset microalbuminuria in type 2 diabetes: A post hoc analysis of the BENEDICT trial. J. Am. Soc. Nephrol..

[33] Furberg CD (2002). Major outcomes in high-risk hypertensive patients randomized to angiotensin-converting enzyme inhibitor or calcium channel blocker vs diuretic: The antihypertensive and lipid-lowering treatment to prevent heart attack trial (ALLHAT). J. Am. Med. Assoc..

[34] Chia YC, Gray SYW, Ching SM, Lim HM, Chinna K (2015). Validation of the Framingham general cardiovascular risk score in a multiethnic Asian population: A retrospective cohort study. BMJ Open.

